# Glucose Metabolism, Thyroid Function, and Prolactin Level in Adolescent Patients With First Episode of Schizophrenia and Affective Disorders

**DOI:** 10.3389/fpsyt.2020.00775

**Published:** 2020-08-05

**Authors:** Maria Giuseppina Petruzzelli, Lucia Marzulli, Orazio Valerio Giannico, Flora Furente, Mariella Margari, Emilia Matera, Francesco Margari

**Affiliations:** ^1^ Department of Basic Medical Sciences, Neuroscience and Sense Organs, University of Bari “Aldo Moro”, Bari, Italy; ^2^ Department of Biomedical Sciences and Human Oncology, University of Bari “Aldo Moro”, Bari, Italy; ^3^ Child Neuropsychiatry Unit, Azienda Ospedaliero-Universitaria Policlinico di Bari, Bari, Italy

**Keywords:** early onset psychopathology, neuroendocrine dysregulation, metabolic syndrome risk factors, insulin resistance, mood disorders, first episode of psychosis (FEP)

## Abstract

Schizophrenia and affective spectrum disorders (ASD) typically begin in adolescence or early adulthood. The pathophysiological mechanisms underlying these disorders are still not fully understood, and recent studies have suggested an involvement of dysfunctions in cardiometabolic and neuroendocrine systems at the onset of both disorders. In this context, we aimed to assess thyroid function, prolactin level, glucose metabolism, and lipid profile in drug naive adolescents, comparing patients with first episode of schizophrenia spectrum disorders (SSD) and patients with ASD. We performed a retrospective chart review from inpatients aged from ten to eighteen years, referred to Child and Adolescent Psychiatric Unit of University of Bari “Aldo Moro” over a period of 4 years, with diagnosis of SSD (n=30) or ASD (n=22), according to Diagnostic and Statistical Manual for Mental Disorders-fifth edition (DSM-5) criteria. Data on serum prolactin, glucose, insulin, total cholesterol, high density lipoprotein cholesterol, low density lipoprotein cholesterol, triglycerides, thyroid stimulating hormone, free triiodothyronin, and free thyroxin were collected, and the insulin resistance (IR) indexes “HOMA1-IR“ and “HOMA2-IR” were calculated. The multivariable linear regression models, adjusting for potential confounding factors (age, sex, and BMI), showed HOMA1-IR (p=0.001), HOMA2-IR (p=0.002), glucose (p=0.004), insulin (p=0.004) and free thyroxin (p<0.001) values higher in the SSD group than in ASD. No others significant differences were found. Our findings suggest the need for a metabolic and endocrine screening at the onset of SSD and ASD, particularly for indexes of IR, that is a testable and treatable risk factor for cardiometabolic diseases. Further studies are required to better understand the role of endocrinological and metabolic dysfunctions at the onset of severe mental illness also considering influencing factors as age, gender, and BMI.

## Introduction

Schizophrenia and bipolar spectrum disorders are considered as part of the psychosis continuum, with similar clinical features such as psychotic and mood symptoms as well as neurocognitive impairments of varying degrees (1). Similarities and differences between neurodevelopmental trajectories in patients with early onset schizophrenia and early onset bipolar disorder have been described with regard to genetic, neurobiological, and environmental risk factors as well as premorbid developmental impairments (2–4). The etiopathological mechanisms underlying both disorders are still not fully understood, although the hypothesis of complex and multifactorial interactions between genetic and environmental risk factors is now widely accepted (2, 3).

Epidemiological studies have clarified that both disorders typically begins in adolescence or early adulthood (5, 6). The relationship between typical changes in the adolescent maturational brain and the full onset of psychopathology is not a unitary phenomenon and one of the fields of greatest interest in this topic is the potential role of hormones in modulating neuronal activity. A lot of evidence supported the association between abnormal gonadal and adrenal hormones levels and different psychopathological conditions (7), anyway, other hormones are thought to have a role in the development and correct functioning of the central nervous system. Recent studies showing dysfunctions in cardiometabolic and neuroendocrine systems, suggested that both psychotic and affective disorders may involve multiple systems at different stage of their clinical course (8, 9).

An increasing number of observational studies on antipsychotic-naïve patients suggested the existence of a pre-diabetic condition at the onset of the psychotic illness, while data from patients with chronic course of schizophrenia showed a higher rate of comorbid metabolic syndrome and type 2 diabetes (10–12). Studies on glucose and lipid metabolism deregulation at the onset of depressive and bipolar disorders are fewer and less agree (13–15). Anyway a bidirectional relationship between major depressive disorder, bipolar disorders, and cardiovascular disease has been proposed (16, 17). Insulin signaling is suggested to play a central role in the mechanisms underlying the association between schizophrenia spectrum disorders (SSD)/affective spectrum disorders (ASD) and cardiovascular risk factors, also considering the potential action of insulin as neuropeptide (18, 19).

Moreover the regulation of glucose homeostasis and insulin sensitivity could be influenced by prolactin (PRL) and thyroid hormones actions (20, 21), with a likely age-dependent variability (22–24). We know that PRL, beside the lactogenic activity, is involved in appetite regulation and plays metabolic actions in both pancreatic and adipose tissue (25), so that hyperprolactinemia (HPRL) could take part in metabolic disorders. Moreover, higher PRL levels have been found in first episode drug-naïve psychotic patients compared to healthy controls; further researches are needed to clarify the relationship between stress, HPRL, and emergence of the psychotic symptoms, also considering the role of confounding factors as age, sex, body mass index (BMI), and thyroid stimulating hormone (26–28). Thyroid dysfunctions are frequently associated in clinical practice with metabolic syndrome (29, 30) as well as a relationship exists between HPRL and hypothyroidism, also in children (31). In addition, altered hypothalamic-pituitary-thyroid system’s function has been described in schizophrenia, bipolar and depressive disorders (32–34), but very few studies have been conducted at the onset of these illnesses (1, 35).

The study of cardiometabolic and neuroendocrine dysfunctions occurring in the acute phase of psychopathological onset may be very informative of their implications in the pathogenesis of SSD/ASD, since some confounding factors related to the chronicity, as persistent negative symptoms, long-term antipsychotic treatment or unhealthy lifestyle, are minimized. Moreover, subjects in adolescent age may be considered at lower risk of cardiovascular disease and endocrine disorders than adult subjects, therefore more suitable to verify the hypothesis of an intrinsic relationship between endocrine-metabolic dysfunctions and psychiatric disorders, despite the limit of larger diagnostic instability and stress-related hormonal variability than in adult patients.

Starting from the hypothesis of a co-shared vulnerability between impaired glucose tolerance and SSD, detectable in subclinical form even in patients with adolescent onset of psychosis, in our previous study we found higher level of PRL and increase in Homeostatic Model Assessment for Insulin Resistance (HOMA-IR) in a sample of drug naïve adolescents in the acute phase of first episode psychosis compared to subjects at clinical high risk of developing psychosis (36). The purpose of the present paper was to extend our previous finding exploring glucose and lipid metabolism as well as PRL regulation and thyroid status, to verify the hypothesis that a greater impairment of the parameters under study may be associated to the adolescent onset of SSD more than of ASD.

Therefore, the aims of the present study were 1. to perform a baseline evaluation of glucose metabolism and lipid profile, PRL level and thyroid function in two samples of drug naïve adolescents in the acute phase of first episode ASD and SSD; 2. to compare the parameters of study between these two different diagnostic groups, adjusting for age, sex and BMI.

## Methods

### Subjects

We performed a retrospective chart review from inpatients of both sexes aged between 10 and 18, referred to Child and Adolescent Psychiatric Unit, Department of Basic Medical Sciences, Neurosciences and Sense Organs over a period of 4 years. According to the purpose of this study, we selected patients that, at the time of admission, had received diagnosis of first episode of SSD or first episode of ASD and had undergone biochemical evaluation of glucose and lipid profiles, PRL level, and thyroid function. Diagnoses of early onset first-episode SSD (schizophrenia, schizophreniform disorder, schizoaffective disorder, psychosis not otherwise specified) were made in accordance to Diagnostic and Statistical Manual for Mental Disorders-fifth edition (DSM-5) criteria (37). The validated Italian version of the Positive and Negative Syndrome Scale (PANSS) (38), was performed within the first 72 h after the admission to assess the severity of psychotic symptoms (positive, negative and general symptoms). Diagnoses of early onset ASD (Bipolar I disorder, Bipolar II disorder, Cyclothymic Disorder, Disruptive Mood Dysregulating Disorder, Major Depressive disorder, Dysthymia) were made in accordance to DSM-5 criteria (37). The validated Italian version of the Hamilton Depression Rating Scale (HAM-D) (39) and Young Mania Rating Scale (YMRS) (40) were performed within the first 72 h after the admission to assess the severity of affective symptoms (depressive, manic, and hypomanic symptoms). Parents and patients were interviewed by two experienced psychiatrists belonging to the research group and the evaluations were discussed in regular reliability meetings, under the supervision of a senior researcher. All the procedures above described were conducted at the time of admission, as part of a more general clinical and laboratory assessment needed for diagnostic evaluation. Patients were excluded: if they were younger than 10 years or older than 18 years; if they had an history of antipsychotics, antidepressants or mood stabilizing assumption; if medical history, physical examination, laboratory, and instrumental findings had revealed that psychopathological symptoms were substance induced or due to another medical condition; if there were any evidences of medical causes of HPRL (such as pituitary/hypothalamic disorders, primary hypothyroidism, renal, and liver insufficiency), abnormal thyroid function and insulin resistance (IR). For each study participant, body weight (kg) and height (m) were measured simultaneously with the blood test; the BMI was obtained by dividing weight by height squared (kg/m^2^). Electrocardiogram, electroencephalogram, and brain magnetic resonance have been used when indicated. Written informed consent from the parents of all participants was obtained during hospitalization so the clinical and laboratory data collected could be used for the research purposes. The approval of methodology of the study was obtained from the independent ethical committee of the University-Hospital Policlinico of Bari.

### Biochemical Measurements

#### Glucose Metabolism Parameters

Peripheral blood samples from all participants were collected between 7,30 and 9 AM, following an overnight fast. Serum glucose was determined using an enzymatic method; levels between 3.33 and 5.55 mmol/L were considered normal for both males and females. Serum insulin was estimated by chemiluminescence. Hyperinsulinemia was defined as values higher than 113.2 pmol/L, for both sexes. HOMA1-IR was calculated using the homeostatic model of assessment as the product of the fasting plasma insulin level (μU/ml) and the fasting plasma glucose level (mmol/L), divided by 22.5. A HOMA1-IR value higher than 2.6 was considered indicative of increased risk of IR, according to references on normal weight adolescents (41–43). We performed the evaluation of HOMA2-IR using the HOMA-2 calculator, version 2.2.3, provided by Oxford University (free download is available from the website www.dtu.ox.ac.uk. No defined thresholds for “normal” vs “abnormal” values are reported).

#### Lipid Profile

Total cholesterol levels were measured through a standardized method traceable to the International Federation of Clinical Chemistry Working Group (IFCC-WG) Reference Method. Levels of 4.03± 0.13 mmol/L for males and 4.47 ± 0.12 mmol/L for females were considered normal. High-density lipoprotein cholesterol (HDLc) was estimated by clearance assay. Levels between 1.01 ± 0.04 mmol/L for males and 1.16 ± 0.03 mmol/L for females were considered normal. Fasting plasma levels of low-density lipoprotein cholesterol (LDLc) were determined using Friedewald formula (44). LDLc levels of 2.61 ± 0.12 mmol/L for males and 2.79 ± 0.1 mmol/L for females were considered normal. Triglycerides (Tg) levels were measured using a traceable IFCC standardized method. Levels within the range 0.25–1.56 mmol/L were considered normal for both male and female patients. Cut point values for acceptable, borderline-high, and high plasma lipid have been considered according to the 2011 Expert Panel on Integrated Guidelines for Cardiovascular Health and Risk Reduction in Children and Adolescents (45).

#### Serum PRL and Thyroid Status

Serum PRL levels were estimated by an immunoassay system. HPRL was defined as PRL levels higher than 0.87 nmol/L in male patients and 1.09 nmol/L in female patients. Thyroid stimulating hormone (TSH) was determined by chemiluminescence; levels between 0.36–3.74 mUI/L were considered normal for both sexes. Free triiodothyronine (fT3) levels were determined by chemiluminescence. Levels between 2.6–8 pmol/L were considered normal for both males and females. Free thyroxin (fT4) was evaluated by chemiluminescence. Levels between 9.78–18.79 pmol/L were considered normal for both sexes.

### Statistical Analyses

Statistical analysis was performed using R 3.5.2 (released on 2018-12-20). Statistical significance α was fixed to 0.05. Categorical variables were reported as absolute and relative frequencies (%) and compared through chi-square test. Numerical variables were reported as mean ± standard deviation and compare through Welch t-test. In order to account for non-normality, evaluated through Shapiro Wilk test, right-skewed numerical variables were transformed in their natural logarithm. To analyze the association between the SSD or ASD and the logarithmic transformation of the 12 parameters, adjusting for potential confounding factors (age, sex, and BMI), 12 multivariable linear regression models were fitted with estimation of the ß coefficients. For each model, a global validation linear model assumption significance test was performed in order to verify the linearity assumption of the dependent variable and the normality and homoskedasticity assumptions of the residuals. We reported all p values and confidence intervals. Considering the features of the working hypotheses, no correction for multiple testing were applied. However, this should be considered in the interpretation of the statistical significance.

## Results

The two samples of study were composed by 30 patients for the SSD group and 22 patients for the ASD group. No significant differences emerged for age, gender and BMI and the mean value of BMI was within normal weight range in both study groups. Demographic and clinical features of the two groups are summarized in **Table 1**. When we performed the multivariate regression analysis, adjusting for potential confounding factors (age, sex, and BMI) (**Table 2**), in order to study the association between the SSD or ASD and the hormonal and metabolic parameters, we found a significant differences concerning HOMA1-IR and HOMA2-IR index, fasting glucose, insulin and fT4 (p values 0.001, 0.002, 0.004, 0.004, <0.001 respectively). Specifically, the HOMA1-IR was significantly higher in the SSD group (3.1 ± 2.0) rather than in ASD group (1.9 ± 1.2), with a mean value indicative of increase risk of IR; also the HOMA2-IR was higher in the SSD group (1.9 ± 1.0) rather than in the ASD group (1.3 ± 0.7). The mean value of fasting glucose, although within the normal range, was significantly higher in SSD group (85.1 ± 12.1) rather than in ASD group (75.9 ± 6.9). In the same way, the mean value of insulin was within the normal range for both groups, but significantly higher in SSD group (14.6 ± 8.7) rather than in ASD group (10.3 ± 6.1). No differences were found between the two groups of study regarding the comparison of lipid profile. Lower mean value of fT4 was found in ASD group (1.0 ± 0.1), with a significant difference when compared with SSD group (1.2 ± 0.2). No significant differences were found for the mean serum level of fT3 and TSH, within the range of normality in both groups. No difference was found between the two groups for the mean serum level of PRL, even if patients with SSD tend to have higher PRL values (24.5 ± 27.6) than patients with ASD (17 ± 12.7), with a mean level near to a condition of HPRL.

**Table 1:** Demographic and clinical features of ASD and SSD patients.

	ASD (n = 22)	SSD (*n* = 30)	P value
**Age [years]** **mean ( S.D.)**	15.4 (1.7)	15.8 (1.3)	0.362
**Gender**			0.235
Male n (%)	6 (27.3)	13 (43.3)	–
Female n (%)	16 (72.7)	17 (56.7)	–
**Residence**	South of Italy	South of Italy	–
**BMI [kg/m^2]** **mean ( S.D.)**	23.2 (5.3)	20.7 (2.7)	0.052
**PANSS mean ( S.D.)** **total** **positive** **negative** **general**	– – – –	91.14 (8.7) 19.28 (2.9) 23 (3.4) 48.85 (4.4)	
**YMRS mean ( S.D.)** **HAMD mean (S.D.)**	32.7 (8) 18.58 (6.6)	– –	– –

ASD, affective spectrum disorder; BMI, body mass index; SSD, schizophrenia spectrum disorder.

**Table 2:** Results of the multivariate regression analysis (p-value), with adjustment by age, sex, and BMI.

	ASD (n = 22)		SSD (*n* = 30)		ß Coefficient	[95%CI]	p value (<0,05)
	Mean value (SD)	Natural logarithmictransformation	Mean value (SD)	Natural logarithmic transformation			
**PRL** **(nmol/L)**	0.7 (0.6)	−0.5 (0.7)	1.1 (1.2)^a^	−0.4 (0.9)	0.18	[−0.26;0.63]	0.407
**TSH** **(mUI/L)**	2.2 (1.3)	0.6 (0.6)	2.0 (1.1)	0.6 (0.5)	0.01	[−0.33;0.35]	0.940
**fT3 (pmol/L)**	4.6(0.7)	1.5 (0.1)	4.7 (0.8)	1.5 (0.2)	0.04	[−0.04:0.13]	0.329
**fT4** **(pmol/L)**	12.6 (1.7)	2.5 (0.1)	15.3 (2.6)	2.7 (0.2)	0.20	[0.10;0.30]	**<0.001**
**Cholester** **(mmol/L)**	3.7 (0.9)^b^	1.3 (0.2)	3.8 (0.7)	1.3 (0.2)	0.03	[−0.1;0.16]	0.629
**LDLc** **(mmol/L)**	2.0 (0.8)^b^	0.6 (0.4)	2.1 (0.6)^a^	0.7 (0.3)	0.1	[−0.15;0.31]	0.499
**HDLc** **(mmol/L)**	1.3 (0.3)^b^	0.2 (0.2)	1.4 (0.3)^a^	0.3 (0.2)	0.11	[−0.02;0.23]	0.086
**Tg** **(mmol/L)**	0.9 (0.4)^b^	−0.3 (0.5)	0.7 (0.3)	−0.4 (0.4)	−0.19	[−0.47;0.09]	0.189
**HOMA2-IR**	1.3 (0.7)	0.1 (0.5)	1.9 (1.0)	0.5 (0.6)	0.53	[0.21;0.85]	**0.002**
**HOMA1-IR**	1.9 (1.2)	0.5 (0.6)	3.1 (2.0)	0.9 (0.7)	0.65	[0.27;1.03]	**0.001**
**Glucose** **mmol/L**	4.2 (0.4)	1.4 (0.1)	4.7 (0.7)	1.5 (0.1)	0.11	[0.04;0.19]	**0.004**
**Insulin** **(pmol/L)**	70.5 (43.0)	4.1 (0.7)	101.1 (60.2)	4.4 (0.7)	0.57	[0.22;0.92]	**0.002**

^a^Missing values for one participant, ^b^Missing values for two participants.

ASD, affective spectrum disorder; fT3, free triiodothyronine; fT4, free thyroxin; HDLc, high-density lipoprotein cholesterol; LDLc, low-density lipoprotein cholesterol; PRL, prolactin; SSD, schizophrenia spectrum disorder; Tg, triglycerides; TSH, thyroid stimulating hormone.

## Discussion

### Glucose and Lipid Metabolism

The main findings of this study were a significant increase in fasting glucose, fasting insulin, and HOMA-IR indexes in drug naïve adolescents with first episode of SSD compared to ones with first episode of ASD, adjusted for age, sex, and BMI. Similar lipid profile was found between the two groups, with mean values including in the acceptable range according to the 2011 Expert Panel on Integrated Guidelines for Cardiovascular Health and Risk Reduction in Children and Adolescents (45). These findings would indicate that adolescents with first episode of SSD might have an increased risk of developing IR and diabetes mellitus compared to adolescents with first episode of ASD, regardless to antipsychotic treatment.

To our knowledge most of the studies on cardiovascular risk factors at the onset of psychiatric illness involved adult patients, with substantial numbers regarding the first episode of SSD more than ASD. Two recent papers comparing cardiovascular risk factors between adolescent samples with first episode of psychosis (FEP) and healthy controls, found that abnormalities in lipid profile resulted associated with early-onset psychosis more than alterations of glucose homeostasis, regardless of antipsychotic treatment. Wedervang-Resell K. et al. reported significantly higher TC/HDLc and Tg values in patients with early onset psychosis, with and without antipsychotic exposure, than healthy subjects; significantly increased in HOMA-IR scores were found only in antipsychotic-exposed patients (46). In the study of Jensen K.G. et al. youth patients with FEP had higher cholesterol and LDLc than matched controls, while increased in insulin and HOMA-IR were found in early onset patients with dyslipidemia or family history of type 2 diabetes mellitus (T2DM) (47). Cardiometabolic risk assessment performed in a sample of recent onset bipolar disorder ranged between 12 and 35 years of age, showed higher triglyceride levels than healthy controls (14).

The variability of these results among studies on adolescent samples may be related to a lot of confounding factors, including dietary intake, sedentary lifestyle, substance use, ethnicity as well as to small samples size and age and sex distribution across different samples of study. Furthermore, an age-related diagnostic heterogeneity could explain some discrepancy between different data, so that longitudinal studies will be more useful to understand the trajectories of changes in glucose tolerance and lipid profile over the time in adolescent population.

Anyway, our hypothesis that adolescents in acute phase of first episode of SSD may have an intrinsic risk of IR appears in accordance with data emerged by systematic review and meta-analysis performed by A.M. Greenhalgh et al. to assess glucose tolerance, insulin and IR in early adulthood antipsychotic-naïve patients with non-affective psychosis (10). Their results showed that, at the time of the onset of psychosis, patients have a slight increase in fasting glucose, usually in the normal range, despite a small increase in IR, by secreting additional insulin (10). In addition, a systematic review and meta-analysis examining lipid parameters in adult patients found that FEP was associated with decreased total and LDLc levels but increased triglyceride levels compared with healthy control groups, with no difference in HDLc levels. The authors suggested that hypertriglyceridemia may be added to the evidence for glucose dysregulation in this cohort, considering it as a feature of T2DM (8). On the other hand IR has been reported in more than half of all bipolar patients and some authors supported the hypothesis that it is associated with the chronic course of illness rather than to early stage (19).

We know that a lot of physiological conditions and disease states, involving neuroendocrine response to stress, were found to be accompanied by IR (48). Increasing evidence showed that a co-shared genetic pathway partially explain the comorbidity of schizophrenia, major depressive disorder, type 2 diabetes, and metabolic syndrome (49, 50) and recent evidence supported the hypothesis that intrinsic dysfunction in central nervous system insulin signaling might represent the final common pathway of interaction between metabolic syndrome schizophrenia and mood disorders (18). As a result, considering that IR may be a reversible condition, the use of HOMA-IR index at the onset of psychosis may be a useful instrument to assess a latent risk of later development of cardiovascular disorders, also in normal weight adolescents (11, 51).

The calculation of HOMA-IR index is a good sensitive and specific method for assessing insulin sensitivity, well accepted by researchers, and used in epidemiological studies in adults, adolescents, and children. The successful application of HOMA-IR index in a given population is related to the use of specific cutoffs for gender, ethnicity, age, and/or sexual maturation level. There is no consensus regarding the reference value of HOMA-IR for the diagnosis of IR in the pediatric age group and several cutoff points have been reported in the literature (43, 52). Moreover, the HOMA index may be assessed using different methods, not exactly comparable, as the original model for the HOMA1-IR or the update HOMA computer model, with some physiological adjustment, for the HOMA 2- IR. It is generally accepted that a value of HOMA1-IR ≥ 2.6 accurately classify normal-weight adolescents at increased cardiovascular and metabolic risk (41–43). According to this indication, the mean value of HOMA1-IR we found in the group of SSD (3.1 ± 2.0) suggested a condition of risk of IR at the onset of psychosis, despite the normal value of other metabolic parameters and BMI. No clear cutoff points have been identified for the use of HOMA2-IR in adolescents, anyway also when we performed the comparison of HOMA2-IR between the two groups of study we found a significant higher mean value in SSD group, suggesting a higher risk of IR in adolescent with SSD rather than with ASD.

#### PRL Levels

Although no significant difference emerged comparing patients with SSD and patients with ASD, we observed a higher mean value of PRL in SSD group, close enough to the cutoff for HPRL. Previous studies have found PRL levels above the physiological limits in first episode drug-naïve psychotic patients (26, 36, 53–55). Riecher-Rossler has suggested that stress may induce HPRL and that both inflammation and deregulation in the serotoninergic system could contribute to the HPRL observed in first episode psychotic patients who have not previously received antipsychotic treatment (54). Future studies evaluating the levels of PRL in drug-naïve patients are needed also considering the role of other factors as hormonal influence, age and gender.

#### Thyroid Status

Regarding evaluation of thyroid function, first of all we must consider that values compatible with good functionality have emerged in both study groups. We know that thyroid disease can always be ruled out when the serum TSH level is normal without drug administration or in the absence of obvious hypothalamic-pituitary disease (56). Anyway, we found a significant lower level of fT4 in ASD group, compared with SSD group and the meaning of this data is not easy to explain. One recent study investigated the association of thyroid function and suicide attempt in major depressive disorder (MDD) patients, showing a lower serum fT4 level in suicide attempters than non-attempters, but without significant differences in TSH and fT3 levels (57). Further studies are needed to clarify the association between thyroid dysfunction and onset of psychotic and affective disorders (56).

### Limitations

Some methodological limitations should be recognized. The small sample size as well as the retrospective design of the study limit the statistical power of the study and, consequently, the generalizability of our results. Moreover, a comparison with a healthy sample would give more value to these findings. Further researches with larger sample size allow us to better characterize abnormalities of hormonal and metabolic parameters with more specific association with diagnostic subgroups. It is important to note that because of the cross-sectional design of the study we cannot infer information about the causality of the relationship between glucose metabolism abnormalities and early onset SSD. Despite these limitations, in our knowledge this is one of the very few studies in this field carried out on a sample of patients under 18.

### Conclusions

In conclusion this study showed an increase in HOMA-IR and higher glucose, insulin levels and free thyroxin in drug naive adolescents with first episode of SSD rather than in first episode of ASD, suggesting the need to perform a metabolic and endocrine screening at the onset of serious mental illness. IR is a testable and treatable modifying factor and early identification may be very important for prevention and management of the progression of cardiometabolic diseases. Further studies with larger sample size and with longitudinal design are needed to better understand the role of endocrinological and metabolic dysfunctions at the onset of severe mental illness also considering influencing factors as age, gender and BMI.

## Data Availability Statement

All datasets presented in this study are included in the article/**Supplementary Material**.

## Ethics Statement

The studies involving human participants were reviewed and approved by Comitato Etico Interregionale, University of Bari “Aldo Moro”. Written informed consent to participate in this study was provided by the participants’ legal guardian/next of kin.

## Author Contributions

MP designed the study and drafted the manuscript. LM contributed in the literature searches and analyses and in the enrolment of the patients. FM contributed in the literature searches and in revising critically of the manuscript. OG performed the statistical analysis. FF contributed in the enrolment and assessment of the patients. MM contributed in the literature searches and in critical revising of the manuscript. EM coordinated the study group and has been involved in revising critically the manuscript.

## Conflict of Interest

The authors declare that the research was conducted in the absence of any commercial or financial relationships that could be construed as a potential conflict of interest.

## Abbreviations

SSD, schizophrenia spectrum disorders; ASD, affective spectrum disorders.
